# Effect of menstruation on girls and their schooling, and facilitators of menstrual hygiene management in schools: surveys in government schools in three states in India, 2015

**DOI:** 10.7189/jogh.09.010408

**Published:** 2019-06

**Authors:** Muthusamy Sivakami, Anna Maria van Eijk, Harshad Thakur, Narendra Kakade, Chetan Patil, Sharayu Shinde, Nikita Surani, Ashley Bauman, Garazi Zulaika, Yusuf Kabir, Arun Dobhal, Prathiba Singh, Bharathy Tahiliani, Linda Mason, Kelly T Alexander, Mamita Bora Thakkar, Kayla F Laserson, Penelope A Phillips-Howard

**Affiliations:** 1 School of Health Systems Studies, Tata Institute of Social Sciences, Mumbai, India; 2 Department of Clinical Sciences, Liverpool School of Tropical Medicine (LSTM), Liverpool, UK; 3 Water Sanitation and Hygiene Section, United Nations Children’s Fund, India; 4 Centers for Disease Control and Prevention (CDC) India, Atlanta, Georgia, USA

## Abstract

**Background:**

Lack of menstrual knowledge, poor access to sanitary products and a non-facilitating school environment can make it difficult for girls to attend school. In India, interventions have been developed to reduce the burden of menstruation for school girls by government and non-governmental organizations (NGOs). We sought to identify challenges related to menstruation, and facilitators of menstrual management in schools in three states in India.

**Methods:**

Surveys were conducted among menstruating school girls in class 8-10 (above 12 years of age) of 43 government schools selected through stratified random sampling in three Indian states (Maharashtra, Chhattisgarh, Tamil Nadu) in 2015. For comparison, ten model schools supported by NGOs or UNICEF with a focussed menstrual hygiene education program were selected purposely in the same states to represent the better-case scenario. We examined awareness about menarche, items used for menstruation, and facilitators on girls’ experience of menstruation in regular schools and compared with model schools. Factors associated with school absence during menstruation were explored using multivariate analysis.

**Findings:**

More girls (mean age 14.1 years) were informed about menstruation before menarche in model schools (56%, n = 492) than in regular schools (36%, n = 2072, *P* < 0.001). Girls reported menstruation affected school attendance (6% vs 11% in model vs regular schools respectively, *P* = 0.003) and concentration (40% vs 45%, *P* = 0.1) and was associated with pain (31% vs 38%, *P* = 0.004) and fear of stain or smell (11% vs 16%, *P* = 0.002). About 45% of girls reported using disposable pads in both model and regular schools, but only 55% and 29% of pad-users reported good disposal facilities, respectively (*P* < 0.001). In multivariate analysis, reported absenteeism during menstruation was significantly lower in Tamil Nadu (adjusted prevalence ratio (APR) 95% confidence interval (CI) = 0.24, 0.14-0.40) and Maharashtra (APR 0.56, CI = 0.40-0.77) compared to Chhattisgarh, and halved in model compared to regular schools (APR 0.50, CI = 0.34-0.73). Pain medication in school (APR 0.71, CI = 0.51-0.97) and use of disposable pads (APR 0.57, CI = 0.42-0.77) were associated with lower absenteeism and inadequate sanitary facilities with higher absenteeism during menstruation.

**Conclusions:**

Menstrual hygiene education, accessible sanitary products, pain relief, and adequate sanitary facilities at school would improve the schooling-experience of adolescent girls in India.

To achieve gender equality, it is important that girls can attend and reach their full potential in schools [1]. Inadequate options for menstrual hygiene recently received attention as a barrier to education for girls in low and middle income countries [2]. Studies have noted poor sanitation in schools and lack of access to good quality sanitary products can be associated with lower enrolment in schools, absenteeism, and dropout [3-6]. Inadequate menstrual hygiene can potentially have health consequences such as increased risk of reproductive and urinary tract infections [5,7-11]. The problem of menstrual hygiene is multifaceted; girls need to be aware about menarche and be able to manage their menstruation in an enabling environment with access to hygienic menstrual materials and facilities for changing and disposal of menstrual items at home and school [3,12]. National and international concerns about menstrual hygiene have been spearheaded through water, sanitation, and hygiene (WASH) programs in schools and policy and programming frameworks to improve knowledge and infrastructure to manage menstrual hygiene [13].

According to 2011 census estimates (the latest available census data), 10% of India’s population were female adolescents aged 10-19 years, which translates into approximately 120 million girls [14]. Although menstruation is celebrated in many parts of India, cultural taboos exist which regularly limit girls from activities during menstruation, including religious restrictions, and freedom to leave the house [3,15]. This contributes to negative attitudes toward menstruation among women, placing a considerable physical and psychological burden on young girls [3]. A systematic review of Indian studies estimated that barely half (48%) of adolescent girls in India were aware of menarche before their first menstruation, and had inadequate knowledge when attaining menarche. It also documented that the paucity of safe and hygienic disposal systems for menstrual items was worrisome [3].

The Government of India has recognized the importance of menstrual hygiene to the health, well-being and educational achievements of girls and women, and has developed several programs to improve menstrual hygiene management (MHM) in schools, targeted at improving knowledge, access and disposal of menstrual waste, and improving sanitation in schools, with support from a number of organisations [16]. Some examples include the production and marketing of low cost sanitary pads [17], government subsidized sanitary pads in rural areas [18], school vending machines for sanitary pads and pad incinerators [17], and increasing gender separated toilet facilities [19].

In light of these government initiatives, a study was developed to evaluate progress on menstrual management in schools in India, and to identify facilitators and barriers to menstrual management in Indian schools in 2015. This paper presents data on cross-sectional surveys conducted among girls in a representative sample of government schools in three states in India, and a comparison with “model” schools receiving additional/intense WASH support in the same states, which allowed us to assess if model schools achieved improvements with regards to menstrual management.

## METHODS

### Study population

The study was carried out in the states Chhattisgarh, Maharashtra, and Tamil Nadu, representing the diverse cultural and socio-economic spectrum in India (Table S1 in **Online Supplementary Document**). Chhattisgarh is a state from central India with a predominantly Tribal population with 2.7 million adolescent girls. Maharashtra is a more developed state in the western part of India with 9.9 million adolescent girls. Tamil Nadu is a southern state having one of the highest levels of development with 6.1 million adolescent girls. Tamil Nadu has implemented a free sanitary pad scheme since 2011, making pads free of cost for girls living in rural areas, those in government schools, and new mothers. Girls can receive three packs of pads once every two months, in addition to iron tablets, and may receive education about menstruation from an “aganwadi” (female community health) worker [20]. Similar programs in Maharashtra and Chhattisgarh are less developed.

A total sample size of 1800 adolescent girls (600 girls per state, about 75 girls per school), would be sufficient to measure a state-based prevalence of 50% with 95% confidence interval and 5% of margin error, taking clustering into account and using a design effect of 1.5. Multi-level stratified sampling was used for each state, by first randomly selecting one district in each of the three states. In each of the selected districts, one block was then randomly selected, and then in each of the selected blocks, a list of all schools was prepared in collaboration with the state government education department in the respective districts. In each of these, schools were then randomly selected from all government middle and high schools (regular) after excluding boys’ only schools, solely residential, and private schools (Figure S1 in **Online Supplementary Document**). Adolescent girls above 12 years of age in class (called grade in India) 8-10 (comparable to school year 8-10 in the United States) were selected to maximise the likelihood they had reached menarche and could provide information on menstrual management and water sanitation and hygiene. One class was randomly selected if there was more than one class in grades 8-10. In Tamil Nadu, more schools were included than in the other states because girls were younger and fewer girls had reached menarche compared to the other states. In addition to regular schools, schools that received support on menstrual hygiene from external sources (“better practice” or model schools) to represent the best case scenario in MHM were purposively chosen with the help of the UNICEF team in the respective state to assess if this resulted in significantly better menstrual management practices. In the model schools, external experts (from UNICEF or other NGOs) regularly provided information sessions on puberty, menstrual hygiene and on how to use menstrual pads.

Schools were visited, and meetings were held with the head teachers. Parental consent forms were then distributed by study staff with the help of school staff. Meetings with target girls who had parental consent were conducted to discuss the study and respond to questions before girls assented. Pre-tested structured self-administered questionnaires in the local languages of each state were used to elicit information on the sanitation status of the school, knowledge about menstruation, pre-menarche, menstrual practices and beliefs, and the effect of menstruation on school life. The data collection was carried out from June to December 2015. Three senior research officers supervised the field data collection team who received intensive one-week training before the start of the study.

### Analysis

For this analysis, only girls who reported they had started menstruating were included. We tabulated results for model and regular schools by state for the following themes: awareness about menarche and source of information, menstruation-related restrictions, menstrual absorbents, effect of menstruation on the school experience, and barriers and facilitators of menstrual management at the school level. Missing data was included as a separate category of the variables of interest. Significant differences were explored at the state level and model vs regular schools overall and within states (χ^2^ test). To assess factors associated with school absence during menstruation, we used generalized linear regression with a log link and binomial distribution for multivariate analyses. Poisson regression with a robust variance estimator was used for models which did not converge (Stata v14.2, StataCorp LLC, College Station, USA). The following factors were explored in univariate analysis: age, state, model vs regular school, menstrual item used, education or program on menstrual hygiene in school and factors related to sanitary situation in school. Factors with a *P*-value <0.1 in the univariate model and model vs regular schools as a focus of interest were included in the multivariate model, whereby factors with a *P*-value >0.05 were removed from the multivariate model using backward elimination. Univariate and multivariate models were adjusted for clustering at the school level, and interactions between significant variables were examined.

### Ethical considerations

The study was approved by the Tata Institute of Social Sciences, Mumbai, and the Liverpool School of Tropical Medicine, UK, after fulfilling all the ethical requirements. Participant information sheets, describing the study and the activities involved for study participants were prepared. Written informed consent from the parents and assent from the girls was obtained before the study, in compliance with national and international ethical committee requirements. The survey questionnaire had ID numbers and had no names on it. Consent forms and questionnaires were translated into local languages of the states involved.

## RESULTS

### Characteristics of schools and participating girls

Of the 3617 girls who participated, 2564 (70.9%) reported they had begun menstruating and were included in this analysis. These menstruating girls attended 43 randomly selected regular schools (N = 2072) and 10 model schools (N = 492) in the 3 states (**Table 1**). Over half of schools were co-educational (58%), a third (31%) was girls’ only, and the remaining had some girls and some mixed classes. Girls’ average age was 14.1 years (standard deviation (SD) = 1.1), with girls from Tamil Nadu slightly younger than other states, and girls in model schools slightly older (14.2 *vs*. 14.0 years, *P* < 0.01). Most girls were in grade 10 (51%), while 35% were in grade 9, and 13% in grade 8. Participating girls were mostly Hindu (93%), with 3% Muslims, and 2% other religions.

**Table 1:** Characteristics of participating schools and girls by state and school type, India 2015

	Maharashtra		Chhattisgarh		Tamil Nadu		All 3 states
	**Regular school, n (%)**	**Model school, n (%)**	**Regular school, n (%)**	**Model school, n (%)**	**Regular school, n (%)**	**Model school, n (%)**	**Total, n (%)**
**Characteristics of schools:**							
Number of schools	12	4	12	4	19	2	53
Type of school:							
-Co-education	10	3	10	0	18	0	41
-Girls only	2	1	2	4	1	2	12
Participants:							
-Not menstruating	169 (19.9)	119 (40.6)	143 (16.9)	56 (18.9)	480 (40.1)	50 (37.6)	1017 (28.1)
-Menstruating	664 (78.2)	173 (59.0)	691 (81.5)	236 (79.7)	717 (59.9)	83 (62.4)	2564 (70.9)
-No answer	16 (1.9)	1 (0.3)	14 (1.7)	4 (1.4)	1 (0.1)	0	36 (1.0)
Median number of menstruating participants per school, range	48, 16-109	45, 22-61	50, 14-149	58, 8-112	24, 15-113	42, 24-59	45, 8-149
**Characteristics of school girls (only menstruating girls included):**							
Total number of participants	664	173	691	236	717	83	2564
Average age of participant (SD)*	14.4 (1.0) n = 645	14.2 (0.9) n = 169	14.3 (1.0) n = 685	14.4 (1.1) n = 236	13.5 (0.9) n = 715	13.6 (0.8) n = 83	14.1 (1.1) n = 2533
Grades of participants:†							
-8	97 (14.6)	31 (17.9)	66 (9.6)	27 (11.4)	104 (14.5)	9 (10.8)	334 (13.0)
-9	230 (34.6)	65 (37.6)	225 (32.6)	92 (39.0)	246 (34.3)	26 (31.3)	884 (34.5)
-10	327 (49.3)	73 (42.2)	389 (56.3)	113 (47.9)	366 (51.1)	48 (57.8)	1316 (51.3)
-Missing	10 (1.5)	4 (2.3)	11 (1.6)	4 (1.7)	1 (0.1)	0 (0.0)	30 (1.2)
Religion:‡							
-Hindu	619 (93.2)	128 (74.0)	686 (99.3)	232 (98.3)	641 (89.4)	79 (95.2)	2385 (93.0)
-Muslim	33 (5.0)	11 (6.4)	1 (0.1)	4 (1.7)	31 (4.3)	0 (0.0)	80 (3.1)
-Other§	12 (1.8)	33 (19.1)	0 (0.0)	0 (0.0)	44 (6.1)	4 (4.8)	93 (3.6)
-No answer	0 (0.0)	1 (0.6)	4 (0.6)	0 (0.0)	1 (0.1)	0 (0.0)	6 (0.2)

SD – standard deviation

*P < 0.01 for comparison by state (Tamil Nadu vs schools in the other states) and model schools (mean 14.2, SD = 1.1) vs regular schools (mean 14.0, SD = 1.0, *P* = 0.005, *t* test).

†*P* < 0.05 comparing schools in Chhattisgarh vs schools in the other states, no difference by model schools vs regular schools (χ^2^ test).

‡*P* < 0.001 comparing schools by states and model schools vs regular schools (χ^2^ test).

§Other: Christian, Buddhist, Jain etc.

### Girls’ awareness and knowledge about menstruation

Nearly all (93%) menstruating girls had received some information about menstruation (**Table 2**). Parents or guardians were the major source (68%), with friends the next most reported source. There were striking differences by state and type of school; approximately 1 in 10 girls said teachers were a common source, the highest proportion were in model schools in Tamil Nadu (51%), and lowest in regular schools in Maharashtra (3%). Half (48%) of girls did not hear about menstruation until their first period began; the proportion of girls who were informed before menarche was significantly higher in model schools compared to regular schools (56% vs 36%, *P* < 0.001).

**Table 2:** Girls’ awareness of menarche and source of information by state and school type, India 2015

	Maharashtra		Chhattisgarh		Tamil Nadu		All 3 states
	**Regular school, n (%)**	**Model school, n (%)**	**Regular school, N (%)**	**Model school, n (%)**	**Regular school, n (%)**	**Model school, n (%)**	**Total**
	**N = 664**	**N = 173**	**N = 691**	**N = 236**	**N = 717**	**N = 83**	**N = 2564**
Who informed you about menstruation?*							
Mother, father, caretaker†	598 (90.1)	87 (50.3)	347 (50.2)	128 (54.2)	517 (72.1)	65 (78.3)	1742 (67.9)
Other relative‡	13 (2.0)	2 (1.2)	78 (11.3)	32 (13.6)	55 (7.7)	9 (10.8)	189 (7.4)
Friends§	79 (11.9)	40 (23.1)	233 (33.7)	76 (32.2)	73 (10.2)	32 (38.6)	533 (20.8)
School teacher (lesson/private)‖	20 (3.0)	38 (22.0)	22 (3.2)	18 (7.6)	89 (12.4)	42 (50.6)	229 (8.9)
Other (eg, doctor, warden)‡	10 (1.5)	3 (1.7)	5 (0.7)	4 (1.7)	0 (0.0)	0 (0.0)	22 (0.9)
No one¶	22 (3.1)	4 (2.3)	22 (3.2)	6 (2.5)	35 (4.9)	0 (0.0)	89 (3.5)
No response	6 (0.9)	7 (4.1)	73 (10.6)	0 (0.0)	0 (0.0)	0 (0.0)	86 (3.4)
**When did you learn about menstruation?**§,**	N = 636	N = 162	N = 596	N = 230	N = 682	N = 83	N = 2389
Before start	242 (38.1)	99 (61.1)	257 (43.1)	119 (51.7)	204 (29.9)	50 (60.2)	971 (40.6)
When 1^st^ period	352 (55.4)	52 (32.1)	244 (40.9)	84 (36.5)	393 (57.6)	29 (34.9)	1154 (48.3)
After 1^st^ period	17 (2.7)	6 (3.7)	46 (7.7)	15 (6.5)	51 (7.5)	3 (3.6)	138 (5.8)
No answer	25 (3.9)	5 (3.1)	49 (8.2)	12 (5.2)	34 (5.0)	1 (1.2)	126 (5.3)

*More than one option was allowed.

†*P* < 0.05 for comparison by state, model schools vs regular schools, and in Maharashtra model vs regular school.

‡*P* < 0.05 for comparison by state.

§*P* < 0.05 for comparison by state, model schools vs regular schools, and in Maharashtra and Tamil Nadu model vs regular school.

‖*P* < 0.05 for comparison by state, model schools vs regular schools, and within states model vs regular school.

¶*P* < 0.05 for comparison in Tamil Nadu model vs regular school.

**Among girls who were informed by persons mentioned above about menstruation (so excluding girls who reported to have not been informed about menstruation and girls with no response to the question).

### Cultural taboos and restrictions during menstruation

**Figure 1:**
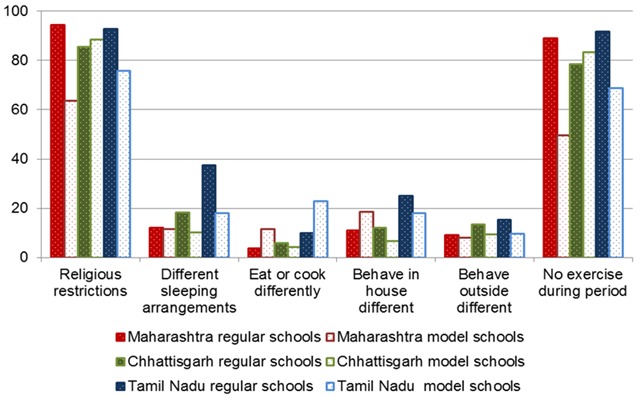
Restrictions (%) during menstruation among school girls in three states in India, 2015. Religious restrictions: *P* < 0.05 comparing model schools vs regular schools, and in Maharashtra and Tamil Nadu model vs regular school. Sleeping arrangements: *P* < 0.05 comparing by state, model vs regular schools, and in Chhattisgarh and Tamil Nadu model vs regular school. Behave different inside house: *P* < 0.05 comparing by state, and in Maharashtra model vs regular school. Behave different outside house: *P* < 0.05 comparing by state, model vs regular school, and in Maharashtra model vs regular school. Eating/exercise: *P* < 0.05 comparing by state, model schools vs regular schools, and in Maharashtra and Tamil Nadu model vs regular school.

Religious restrictions (not going to temple, *etc.*) were common, affecting 88% of girls overall, and nearly all (91%) girls in regular schools (**Figure 1**, and Table S2 in **Online Supplementary Document**); girls reported less restrictions in the model schools in Maharashtra (64%) and Tamil Nadu (76%) compared to the regular schools (94% and 93%, respectively, *P* < 0.001). Restrictions during exercise were prevalent (83% overall); again this was significantly lower in model schools in Maharashtra (89% vs 50%, *P* < 0.001) and Tamil Nadu (92% vs 69%, *P* < 0.001) but not in Chhattisgarh (78% vs 84%, *P* = 0.205). Other reported restrictions due to cultural traditions were less common (**Figure 1**); thus one in five reported different sleeping arrangements, 16% reduced social interactions within the home, 12% reduced social interactions outside the home, and 7% had restricted food choices.

### Items used for menstrual hygiene management by girls

**Figure 2:**
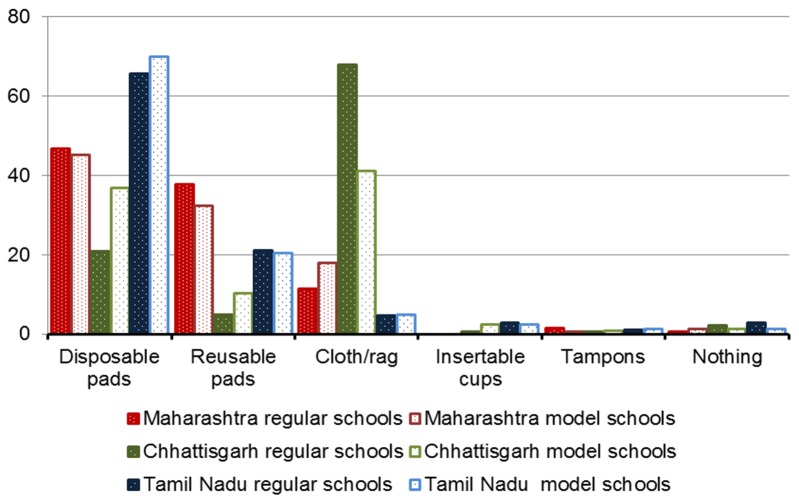
Items (%) used to deal with menstruation in three states in India, 2015. Disposable pads: *P* < 0.05 by state, and in Chhattisgarh model vs regular schools. Reusable pads: *P* < 0.05 by state, and in Chhattisgarh model vs regular schools. Cloth/rag: *P* < 0.05 by state, and in Chhattisgarh model vs regular schools. Tampon: no differences. Cup: *P* < 0.05 by state, and in Chhattisgarh model vs regular schools.

Overall, 45% of girls used disposable sanitary pads, 28% used cloths, and 21% reusable pads. Menstrual cups and tampons were reported by 1% each, 2% of girls said they did not use anything, and 3% did not respond. There were considerable differences by state and school (**Figure 2** and Table S3 in **Online Supplementary Document**). The majority of girls in Chhattisgarh used cloths (61%), whereas the majority of girls in Maharashtra and Tamil Nadu used disposable pads (47% and 66%, respectively). Reusable pads were mainly used in Maharashtra (37%) and Tamil Nadu (21%). Only in Chhattisgarh significant differences were present between model and regular schools; cloths were used less frequently in model schools where disposable pads were more common.

### Effect of menstruation on school experience

The majority of girls reported going to school during their menstruation (87%, **Table 3**), and this was higher among model schools (92% vs 86% in regular schools, *P* = 0.003). One out of five girls in regular schools in Chhattisgarh reported missing school during their period. The majority (65%) of girls reporting absence stated it was for 1 day, 22% said 2-3 days and 13% responded that it was throughout menstruation. Concentration problems at school during menstruation were common (45%), with differences noted by state (**Table 3**). Other frequently mentioned problems in school included pain (stomach, head, hips and limbs, 36%), fear of staining or smell or losing the cloth or pad in school (15%), feeling unwell, tired, dizzy, and weak (11%). A few (5%) reported reduced mobility and comfort resulting in problems with sitting, walking, bicycling, and reaching school. Girls who used disposable pads were significantly more likely to report attending school during menstruation (95%), and less frequently reported concentration or other problems during menstruation (39%, and 47%, respectively) than girls who used cloths (81%, 53%, and 68%, respectively, *P* < 0.001 for all comparisons, Figure S2 and S2 in **Online Supplementary Document**).

**Table 3:** Effect of menstruation on school experience

	Maharashtra		Chhattisgarh		Tamil Nadu		All 3 states
	**Regular school, n (%)**	**Model school, n (%)**	**Regular school, n (%)**	**Model school, n (%)**	**Regular school** **n (%)**	**Model school** **n (%)**	**Total**
	N = 664	N = 173	N = 691	N = 236	N = 717	N = 83	N = 2564
Go to school during period:*							
Yes	587 (88.4)	162 (93.6)	520 (75.3)	206 (87.3)	684 (95.4)	82 (98.8)	2241 (87.4)
No	67 (10.1)	7 (4.1)	143 (20.7)	23 (9.8)	25 (3.5)	0	265 (10.3)
No response	10 (1.5)	4 (2.3)	28 (4.1)	7 (3.0)	8 (1.1)	1 (1.2)	58 (2.3)
**Concentration problems at school during menstruation:†**							
Yes	336 (50.6)	68 (39.3)	343 (49.6)	107 (45.3)	263 (36.7)	24 (28.9)	1141 (44.5)
No	316 (47.6)	97 (56.1)	333 (48.2)	125 (53.0)	445 (62.1)	59 (71.1)	1374 (53.6)
No response	12 (1.8)	8 (4.6)	15 (2.2)	4 (1.7)	9 (1.3)	0 (0.0)	48 (1.9)
**Do you have other problems when attending school during menstruation?‡**							
Yes	439 (66.1)	119 (68.8)	477 (69.0)	109 (46.2)	279 (38.9)	15 (18.1)	1438 (56.1)
No	217 (32.7)	40 (23.1)	176 (25.5)	107 (45.3)	431 (60.1)	68 (81.9)	1039 (40.5)
No response	8 (1.2)	14 (8.1)	38 (5.5)	20 (8.5)	7 (1.0)	0 (0.0)	87 (3.4)
**Specification of some problems when attending school during menstruation:**							
Pain during menstruation§	242 (36.5)	69 (40.0)	209 (44.7)	69 (29.2)	228 (31.8)	13 (15.7)	930 (36.3)
Fear of stains, smell, loss of item‖	152 (23.0)	25 (14.5)	125 (18.1)	25 (10.6)	60 (8.4)	2 (2.4)	389 (15.2)
Feeling tired, dizzy, weak, unwell¶	127 (19.1)	38 (22.0)	57 (8.3)	18 (7.6)	46 (6.4)	7 (8.4)	293 (11.4)
Discomfort when moving or sitting¶	24 (3.6)	3 (1.7)	54 (7.8)	23 (9.8)	18 (2.5)	2 (2.4)	124 (4.8)

*P < 0.05 comparing by state, model schools vs regular schools, and in Maharashtra and Chhattisgarh model vs regular school.

†*P* < 0.05 comparing by state, and in Maharashtra model vs regular school.

‡*P* < 0.05 comparing by state, model schools vs regular schools, and within states model schools vs regular schools.

§*P* < 0.05 comparing by state, model schools vs regular schools, and in Chhattisgarh and Tamil Nadu model vs regular school.

‖*P <* .05 comparing by state, model schools vs regular schools, and in Maharashtra and Chhattisgarh model vs regular school.

¶*P* < 0.05 comparing by state.

### Facilitation of schools of menstrual hygiene management

#### Toilet and wash facilities reported by girls

About half of girls thought there were enough toilets in the school to deal with their menstruation, with the lowest proportion in the regular schools in Maharashtra (33%), and the highest in model schools in Tamil Nadu (99%; **Table 4**). Only 37% of girls stated their school had toilets exclusively for them, with the highest proportion in Tamil Nadu (60%). Access to toilets differed by state, with 48% of girls in Chhattisgarh stating they could use them any time, while the majority of girls in other states were only allowed during break-time. For accidental leaking of blood during lessons, a higher proportion of girls in model schools stated they were allowed to leave the class (63% in regular vs 76% in model schools, *P* < 0.001). Most girls thought there was enough time for changing their menstrual item during break (55% in regular and 73% in model schools, *P* < 0.001). Washing facilities in schools were insufficient, with overall just 51% of girls reporting washing was always possible.

**Table 4:** Facilitators by schools of menstrual hygiene management

	Maharashtra		Chhattisgarh		Tamil Nadu		All 3 states
	Regular school, n (%)	Model school, n (%)	Regular school, N (%)	Model school, n (%)	Regular school n (%)	Model school n (%)	Total
	N = 664	N = 173	N = 691	N = 236	N = 717	N = 83	N = 2564
**Are there enough toilets to deal with menstruation in the school?‡**							
Yes	220 (33.1)	104 (60.1)	268 (38.8)	94 (39.8)	494 (68.9)	82 (98.8)	1262 (49.2)
No	399 (60.1)	54 (31.2)	376 (54.4)	128 (54.2)	214 (29.9)	1 (1.2)	1172 (45.7)
No answer	45 (6.8)	15 (8.7)	47 (6.8)	14 (5.9)	9 (1.3)	0	130 (5.1)
**Toilets for girls:§**							
For female staff & girls	166 (25.0)	34 (19.7)	79 (11.4)	95 (40.3)	195 (27.2)	29 (34.9)	598 (23.2)
For girls only	123 (18.5)	47 (27.2)	183 (26.5)	90 (38.1)	441 (61.5)	50 (60.2)	934 (36.5)
For boys and girls	188 (28.3)	61 (35.3)	242 (35.0)	5 (2.1)	62 (8.7)	4 (4.8)	562 (21.9)
For all staff & students	154 (23.2)	15 (9.7)	108 (15.6)	35 (14.8)	11 (1.5)	0	323 (12.6)
No response	33 (5.0)	16 (9.3)	79 (11.4)	11 (4.7)	8 (1.1)	0	147 (5.7)
**When can you use the toilet?‖**							
Any time	215 (32.4)	41 (23.7)	323 (46.7)	125 (53.0)	175 (24.4)	22 (26.5)	901 (35.1)
Only during breaks	395 (59.5)	115 (66.5)	213 (30.8)	75 (31.8)	527 (73.5)	60 (72.3)	1385 (54.0)
Other responses*	30 (4.5)	2 (1.2)	38 (5.5)	4 (1.7)	11 (1.5)	1 (1.2)	86 (3.4)
No response	24 (3.6)	15 (8.7)	117 (16.9)	32 (13.6)	4 (0.6)	0	192 (7.5)
**Is there enough time in breaks for change of menstrual item?‖**							
Yes	307 (46.2)	132 (76.3)	301 (43.6)	147 (62.3)	532 (74.2)	80 (96.4)	1499 (58.5)
No	343 (51.7)	30 (17.3)	342 (49.5)	84 (35.6)	175 (24.4)	3 (3.6)	977 (38.1)
No response	14 (2.1)	11 (6.4)	48 (6.9)	5 (2.1)	10 (1.4)	0	89 (3.4)
**Are you allowed to leave class if leaking?¶**							
Yes	354 (53.3)	133 (76.9)	367 (53.1)	162 (68.6)	593 (82.7)	77 (92.8)	1686 (65.8)
No	295 (44.4)	34 (19.7)	266 (38.5)	64 (27.1)	114 (15.9)	6 (7.2)	779 (30.4)
No response	15 (2.3)	6 (3.5)	58 (8.4)	10 (4.2)	10 (1.4)	0	99 (3.9)
**Can you wash yourself in school when leaking?‡**							
Can always wash in school	230 (34.6)	131 (75.7)	317 (45.9)	114 (48.3)	443 (61.8)	72 (86.8)	1307 (51.0)
Can sometimes wash	47 (7.1)	12 (6.9)	113 (16.4)	50 (21.2)	209 (29.2)	11 (13.3)	442 (17.2)
Can never wash in school	375 (56.5)	18 (10.4)	219 (31.7)	60 (25.4)	48 (6.7)	0	720 (28.1)
No response	12 (1.8)	12 (6.9)	42 (6.1)	12 (5.1)	17 (2.4)	0	95 (3.7)

*Other included responses such as queuing before toilet, toilet unusable, no toilet present, go home for change

‡*P* < 0.05 for comparison by state, model vs regular school, and Maharashtra and Tamil Nadu model vs regular school.

§*P* < 0.05 for comparison by state, model vs regular school, and Maharashtra and Chhattisgarh model vs regular school.

‖*P <* .05 for comparison by state, model vs regular school, and Maharashtra model vs regular school.

¶*P* < 0.05 for comparison by state, model vs regular school overall and within states.

#### Disposal facilities as reported by girls

**Figure 3:**
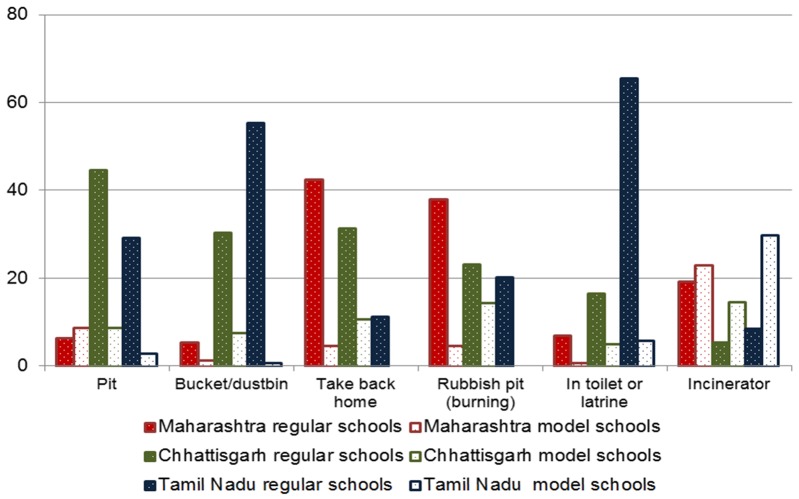
Disposal options (%) of menstrual items in schools in three states in India, 2015. *Excluding participants who used reusable pads or cups. Pit: *P* < 0.05 for comparison by state, and for Maharashtra and Chhattisgarh model vs regular school. Bucket/dustbin: *P* < 0.05 for comparison by state and model vs regular school, and in Tamil Nadu model vs regular school. Take back home: *P* < 0.05 for comparison by state, model vs regular school, and in Maharashtra and Tamil Nadu model vs regular school. Rubbish pit for burning: *P* < 0.05 by state and in Maharashtra, Chhattisgarh, and Tamil Nadu comparing model vs regular schools. In toilet/latrine: *P* < 0.05 by state and type of school. School incinerator: *P* < 0.05 by state, type of school, and in Maharashtra, Chhattisgarh and Tamil Nadu model vs regular school.

Only 27% of girls reported that their schools had good disposal facilities for menstrual waste, and options varied widely across states and schools (**Figure 3**, and Table S4 in **Online Supplementary Document**). The most frequently mentioned option for disposal was taking the soiled item home (21%), with 41% of girls in regular schools in Maharashtra saying this. Burn pits (20%), rubbish pits (17%), or bins (16%) were the next most common, with a low proportion (7%) reporting an incinerator for waste. Incinerators were more common in model schools; for example 2% of girls in Tamil Nadu regular schools reported incinerators, whereas this was 64% in model schools (*P* < 0.001). In Tamil Nadu, 19% of girls reported throwing menstrual waste down the toilets, compared with <5% in the other two states where free napkins were less available. When limiting analysis specifically to the 1153 girls using disposable pads, 37% reported disposal in burning or rubbish pits, 17% in buckets, 9% in an incinerator, 11% in toilets, and 19% reported taking the used pad home.

#### Additional facilitation (pain relief, pad provision, point person for menstrual hygiene management)

**Figure 4:**
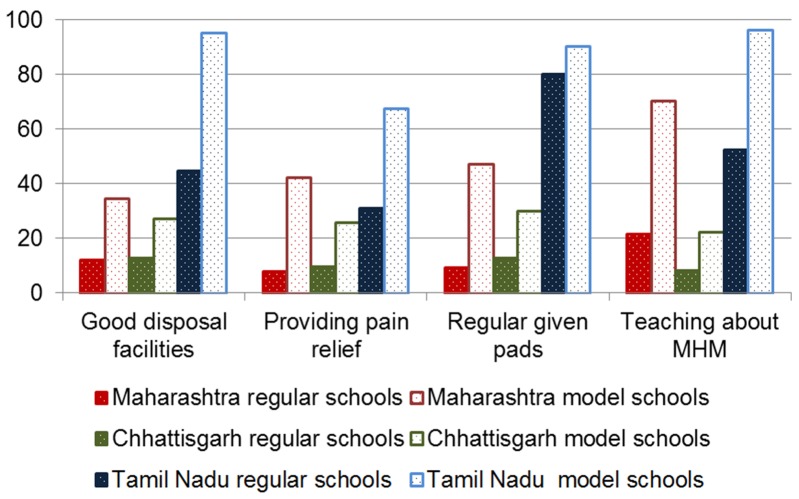
Facilitators of menstrual hygiene (%) in schools in three states in India. Good disposal facilities: *P* < 0.05 for comparison by state, model vs regular school overall and within states. Providing pain relief: *P* < 0.05 for comparison by state, model vs regular school overall and within states. Regularly given pads: *P* < 0.05 for comparison by state, model vs regular school overall and within states. Teaching about menstrual hygiene: *P* < 0.05 for comparison by state, model vs regular school overall and within states.

Overall, 21% of girls reported they could get pain relievers for menstrual cramps in the school when needed, with a significantly higher proportion in model (39%) compared with regular schools (17%) in all states (*P* < 0.001, **Figure 4**, and Table S4 in **Online Supplementary Document**). Overall, 37% of girls said absorbents were made available to them in school. This was almost exclusively due to pad provision in Tamil Nadu, with 81% of girls saying they were regularly given pads. Pad distributions were significantly more common in model schools than in regular schools (overall 46% vs 35%, *P* < 0.001), and, within states, in Maharashtra (47% vs 9%, respectively, *P* < 0.001) and Chhattisgarh (30% vs 13%, respectively, *P* < 0.001). Overall, 51% of girls reported they knew a point person in the school they could approach for problems with menstrual hygiene management, mostly (75%) this was a female teacher (Table S4 in **Online Supplementary Document**).

### Education in schools on menstruation and menstrual hygiene

Overall, 34% of girls reported to have received education about menstrual hygiene in school; the proportion differed significantly by state, type of school and within states (Table S5 in **Online Supplementary Document**). The majority of girls heard about it in a hygiene lesson (58%), during lessons separate from boys (82%). Written materials about menstruation were infrequently available (19%) and mainly present in model schools. Of the 1742 girls who heard about menstrual hygiene from their parents or guardians, 586 (34%) had lessons at school as well.

### Factors associated with missing school during menstruation

Numerous factors were associated with missing school during menstruation in univariate analysis (**Table 5**); however, six remained in the multivariate model. State and type of school affected absence rates, and were significantly lower in Tamil Nadu (adjusted prevalence ratio (APR) = 0.24, 95% confidence interval (CI) = 0.14-0.40) and Maharashtra (APR = 0.56,95% CI = 0.40-0.77) compared to Chhattisgarh, and halved in model compared to regular schools (APR = 0.50, 95% CI = 0.34-0.73) The use of disposable pads, the availability of pain medication, and a space to wash in school were all associated with less absenteeism during menstruation. Dysfunctional toilets or long queues for toilets were associated with increased absenteeism. In a separate multivariate analysis including only variables related to sanitary facilities at school, “clean toilets”, “toilet breaks”, and “can wash in school” remained significant; however, “gender-separate toilets” was not significant in the multivariate analysis (Table S6 in **Online Supplementary Document**).

**Table 5:** Factors associated with missing school during menstruation by adolescent girls, 3 states in India, 2015

Factor	Univariate analysis		Multivariate analysis		
**Missing school: n/N (%)**	Prevalence ratio, 95% CI*	***P*-value**	Prevalence ratio, 95% CI*	***P*-value**
**Age (years):**					
13 and below	47/665 (7.1)	Reference		NS	
14	97/981 (9.9)	1.43, 0.97, 2.10	0.068		
15 and above	118/832 (14.2)	2.03, 1.44-2.85	<0.001		
**State:**					
Chhattisgarh	166/892 (18.6)	Reference		Reference	
Maharashtra	74/823 (9.0)	0.48, 0.31-0.75	0.001	0.56, 0.40-0.77	<0.001
Tamil Nadu	25/791 (3.2)	0.17, 0.10-0.28	<0.001	0.24, 0.14-0.40	<0.001
Model school					
Yes	30/480 (6.3)	0.54, 0.26-1.13	0.104	0.50, 0.34-0.73	<0.001
No	235/2026 (11.6)	Reference			
**Menstrual item used:**					
Nothing or NR	15/94 (16.0)	0.84, 0.49-1.44	0.523	1.19, 0.73-1.95	0.490
Cloth	131/688 (19.0)	Reference		Reference	
Reusable pads	48/525 (9.1)	0.48, 0.32-0.72	<0.001	0.98, 0.74-1.31	0.893
Disposable pads	58/1140 (5.1)	0.27, 0.19-0.39	<0.001	0.57, 0.42-0.77	<0.001
Insertables†	13/59 (22.0)	1.16, 0.70-1.91	0.569	2.51, 1.54-4.07	<0.001
**Pain medication in school:**					
Yes	25/528 (4.7)	0.39, 0.26-0.59	<0.001	0.71, 0.51-0.97	0.031
No or not reported	240/1978 (12.1)	Reference		Reference	
**Pads given in school:**					
Yes	50/943 (5.3)	Reference		NS	
No	181/1329 (13.6)	2.57, 1.74-3.80	<0.001		
Don’t know	17/153 (11.1)	2.10, 1.11-3.95	0.022		
Not reported	17/81 (21.0)	3.96, 2.40-6.54	<0.001		
**Education MH in school:**					
Yes	47/824 (5.7)	Reference		NS	
No	197/1432 (13.8)	2.41, 1.65-3.53	<0.001		
Don’t know	11/188 (5.9)	1.03, 0.57-1.86	0.935		
Not reported	10/62 (16.1)	2.83, 1.43-5.60	0.003		
**MH program in school:**					
Yes	522 (20.4)	Reference			
No	1565 (61.0)	3.01, 1.77-5.12	<0.001	NS	
Don’t know	363 (14.2)	1.51, 0.75-3.02	0.246		
Not reported	114 (4.5)	3.30, 1.54-7.06	<0.001		
**Enough toilets in school:**					
Yes	93/1240 (7.5)	0.55, 0.39-0.78	0.001	NS	
No	156/1153 (13.5)	Reference			
Not reported	16/113 (14.2)	1.05, 0.52-2.09	0.898		
**When can you use the toilet?:**					
Any time	100/887 (11.3)	Reference		Reference	
Only during breaks	106/1365 (7.8)	0.69, 0.49-0.96	0.029	0.95, 0.71-1.27	0.744
Other responses§	21/85 (24.7)	3.18, 1.70-5.95	<0.001	1.61, 0.98-2.66	0.062
No response	38/169 (22.5)	2.90, 1.91-4.38	<0.001	1.42, 1.01-1.99	0.045
**Toilets clean:**					
Always clean	73 (1128 (6.5)	Reference		NS	
Sometimes clean	122/978 (12.5)	1.93, 1.32-2.80	0.001		
Never clean or NR	70/396 (17.7)	2.73, 1.74-4.29	<0.001		
**Toilets for girls:**					
For female staff & girls	60/582 (10.3)	1.40, 0.94-2.07	0.096	NS	
For girls only	68/921 (7.4)	Reference			
For boys and girls	79/549 (14.4)	1.95, 1.07-3.55	0.029		
For all staff & students	38/320 (11.9)	1.61, 0.96-2.71	0.073		
No response	20/134 (14.9)	2.02, 1.10-3.72	0.024		
**Can wash in school:**					
Can always wash	95/1287 (7.4)	Reference		Reference	
Can sometimes wash	47/437 (10.8)	1.46, 1.07-1.98	0.017	1.44, 1.07-1.92	0.015
Can never wash or NR	123/778 (15.8)	2.14, 1.56-2.94	<0.001	1.49, 1.13-1.95	0.004
**Disposal options at school:**					
In pits	92/927 (9.9)	3.07, 0.96-9.85	0.059	NS	
In buckets	44/389 (11.3)	3.47, 1l06-11.36	0.040		
Take home	65/515 (12.6)	3.96, 1.23-12.72	0.021		
Throw in toilet	17/202 (8.4)	2.60, 0.78-8.60	0.119		
Incinerator	6/186 (3.2)	Reference			
Other or no answer	38/259 (14.7)	4.50, 1.29-15.72	0.019		

CI – confidence interval, MH – menstrual hygiene, NR – not reported, NS – not significant

*All analyses adjusted for school as cluster. Factors explored but not significant included class, time of transport to school, and means of transport to school. No interactions of interest were noted between significant variables.

†Tampons or menstrual cups.

§Other included responses such as queuing before toilet, toilet unusable, no toilet present, go home for change.

## DISCUSSION

This study explored the current progress of both government and external agencies to reduce the barriers menstruation causes for schoolgirls in India, and identified where actions can be taken to improve this further. Menstruation was not only shown to impact absenteeism (among 10% of girls) but also affected the quality of school time, with close to half of the girls complaining of an inability to concentrate when in school, and about a third complaining of pain (36%); other worries included fear of staining, smell, or feeling unwell, and discomfort with movement and sitting. These problems were affected by the type of menstrual item used, eg, they were more common among users of cloth (used by 28% of girls) compared to disposable pad users (used by 45%). The status of sanitary facilities was reported to be often inadequate, compromising girls’ ability to manage their menstruation in school. Model schools had half the reported menstrual-related absence, and compared to Chhattisgarh absence was 75% lower in Tamil Nadu where sanitary napkin schemes predominate. Simply providing sanitary pads would clearly not resolve girls’ menstrual issues, however. Comparison of regular against `model’ schools highlighted that additional activities reach girls and improved their knowledge, and ability to cope with menstruation in school. Variations between states displayed a need to tailor interventions to address differing cultural and socio-geographical challenges; eg, in regions where cloths are routinely used, girls would need information on how to hygienically clean and dry them. This study also demonstrated the ongoing need for improving sanitary and disposal facilities at the schools (eg, one in five girls using disposable pads had to take the used napkin home for disposal), and encouraging (development and) use of biodegradable pads. Gains can be achieved from simple measures such as pain relief in school or relaxation of school-break rules.

Most girls were not aware of menarche and faced barriers and restrictions when menstruating, consistent with past studies across India [3,6]. While health education is a common thread across the Government of India schemes, our study found no evidence of menstrual education offered systematically in regular schools. Model schools’ focused programmes significantly improved girls’ awareness of menstrual hygiene suggesting this provides a template to reframe girls’ understanding that menstruation is a normal physiological process [21,22]. However, parents were the main source of information about menstruation, and efforts to equip families with information to prepare daughters on menarche and menstrual hygiene would add value to school-based initiatives, and are included in government guidelines [6,23]. While differences exist across states, it was an interesting and encouraging observation that some restrictions related to menstruation were less in model schools (with more attention to menstrual hygiene) compared to regular schools. The education of girls might have a wider effect on family and society. As our study is cross-sectional, it is not possible to clarify this.

Approximately half of girls reported using disposable pads. The high use in Tamil Nadu reflects the implementation of the free sanitary pads scheme in this state [20]; it is notable that about 20% of girls choose to use reusable pads in Tamil Nadu, which may be because of the disposal issues for disposable pads. A preference for reusable pads has also been reported among women living in slums in Hyderabad [24]. The wider reported experience of differing menstrual products suggests other products, currently deemed to be unacceptable due to the many taboos, may indeed be welcomed by girls and women in India. Although girls clearly seem to benefit from a scheme as implemented in Tamil Nadu and envisioned for the rural areas in India [20], inadequate disposal hinders success. Incinerators have been promoted as an option, but there are concerns about the environmental impact [25]. According to solid waste management rules of the Government of India, sanitary pad manufacturers must provide a wrapper with each pad, and must be deposited in landfills as non-biodegradable waste [26,27]. For the environment, the reusable options for menstruation may be preferable; biodegradable menstrual pads, now being tested in India, may be another option. It is unclear if girls are aware of all the options available to deal with menstruation, and very likely that their access to some of them will be limited (eg, reusable pads, menstrual cup, or tampons). Lack of adequate toilet facilities emerges as one of the major reasons for girls’ absenteeism which has been demonstrated by others [3]. While government systems suggest that all schools have enough gender specific toilets [28], girls’ responses do not corroborate this with 46% of girls saying there were not enough toilets, and only 37% saying they were for girls only. Even when toilets were present, the functionality could be doubted when girls responded the toilets were unusable so they preferred to go outside or stay home. In addition, only 35% of the girls reported they could use the toilets anytime (not only during breaks) and only 51% reported they could always wash themselves in school. The time during break was too short for a change of the menstrual item for one third of the girls (38%), and thirty percent responded they could not leave the class when leaking. A more flexible approach of school rules with allowance of toilet visits during lessons may better facilitate girls’ menstrual hygiene and reduce absenteeism. About one third of girls had some form of pain during menstruation; it is encouraging that the ability of provision of pain relief in school may assist in keeping girls in school during their period.

India is a country of contrasts with strong gender-related disparities; a strength of this study is that we adopted the same methodology across three geographical locations. We are aware that this still would not allow us to generalize the findings to government schools in other States of India, or to private schools. Nonetheless, it gives an opportunity to understand menstrual management in-depth with a large sample size across the country. Some girls in the study did not respond to questions, especially in Chhattisgarh; for example, 10% of girls in the overall sample and 20% in Chhattisgarh did not give any response about disposal of menstrual waste in the school. Great care was taken with the use of local words, and questionnaires were pre-tested to get accurate information. Despite our efforts, there is a possibility that some girls had difficulty in understanding some questions, and self-reported responses may suffer from “desirability bias”. Model schools were selected so they may have been prone to bias; however they may illustrate the “best case scenario”. Researchers were not aware of the type of interventions which had occurred in the model schools or regular schools involved. Studies were cross-sectional, so causality cannot be inferred.

In conclusion, our study further strengthens the case for national investment in menstrual hygiene management by schools. Focused national policies and budget support for menstrual hygiene would facilitate schools to improve this in a continuous and sustainable way. Ensuring sufficient gender specific private toilet facilities with water for changing and washing, and provision of sanitary materials would help reduce girls’ absenteeism in schools during menstruation. Providing pain relief, and adapting school rules (to facilitate toilet visits) may further help to facilitate menstrual care in schools. Broader policy implications include the responsiveness of the education sector to enhance girls’ reproductive health and life skills, and modify social norms to diminish menstrual restrictions. International investment in the development of environmentally-friendly materials and disposal systems is also called for.

## Additional Material

Online Supplementary Document
