# Supplementary material for: Effect of menstruation on girls and their schooling, and facilitators of menstrual hygiene management in schools: surveys in government schools in three states in India, 2015
Source: J Glob Health. 2018 Dec 7;9(1):010408. doi: 10.7189/jogh.09.010408 (PMC6286883; doi:10.7189/jogh.09.010408)
Supplement: Online Supplementary Document [file jogh-09-010408-s001.pdf]

Supplement to:

Effect of menstruation on girls and their schooling, and facilitators of menstrual hygiene management in schools: surveys in government schools in three states in India, 2015

CONTENTS

Figure S1: Random sampling for field surveys, 3 Indian states ... 2

Figure S2: Reported school problems related to menstruation by type of menstrual item used, three states in India, 2015 ... 3

Figure S3: Specification of school problems related to menstruation by type of menstrual item used, three states in India, 2015 ... 4

Figure S4: Preferred MHM item by item currently using, government schools in three states in India, 2015 ... 5

Table S1: Summary table overviewing population, economic, educational, health and sanitation indicators in States involved and the whole of India ... 6

Table S2: Girls' behaviour with regards to cultural taboos and restrictions during menstruation by state and school type, India 2015 ... 7

Table S3: Menstrual hygiene item used and preference by adolescent school girls in three States in India, 2015 ... 8

Table S4: Other Facilitators by schools of menstrual hygiene management, government schools in India, 2015 ... 9

Table S5. Education on MHM in schools, surveys in government schools in three states in India, 2015 ... 12

Table S6. The association between sanitation related factors and missing school during menstruation by adolescent girls, 3 states in India, 2015 ... 14

FIGURE S1: RANDOM SAMPLING FOR FIELD SURVEYS, 3 INDIAN STATES

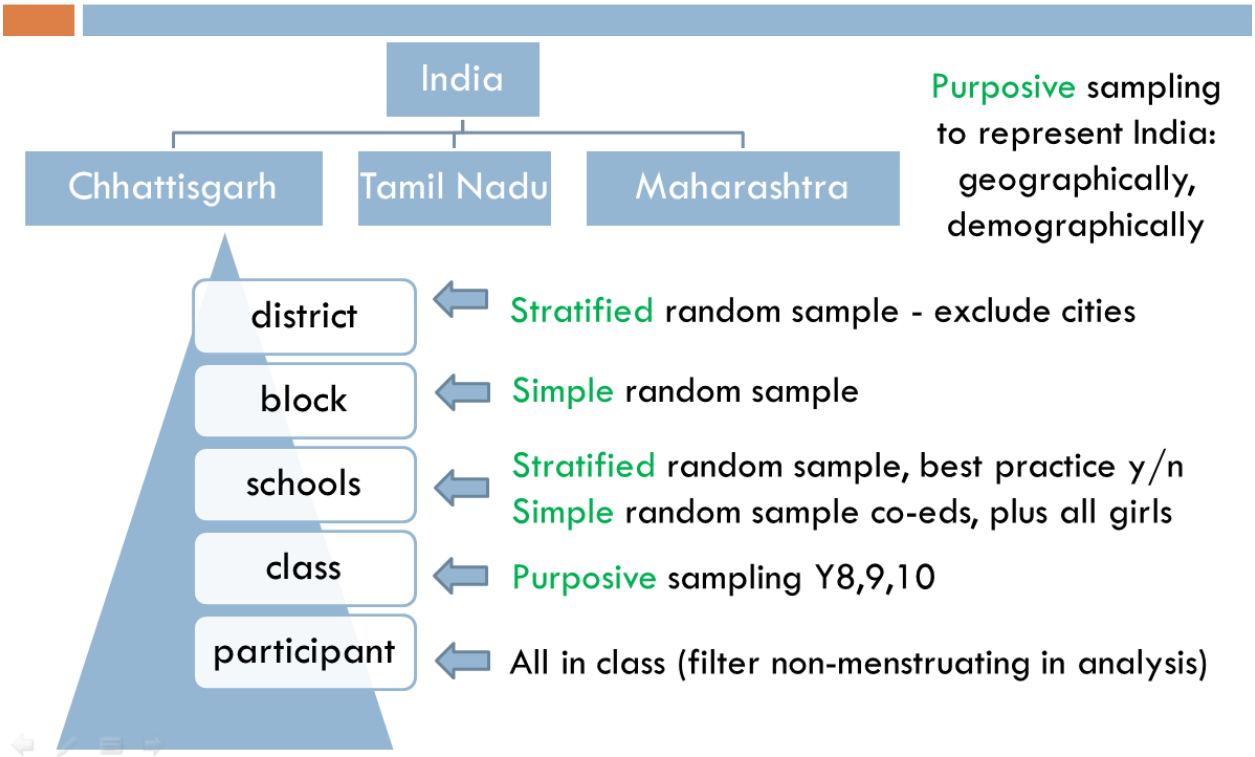

FIGURE S2: REPORTED SCHOOL PROBLEMS RELATED TO MENSTRUATION BY TYPE OF MENSTRUAL ITEM USED, THREE STATES IN INDIA, 2015

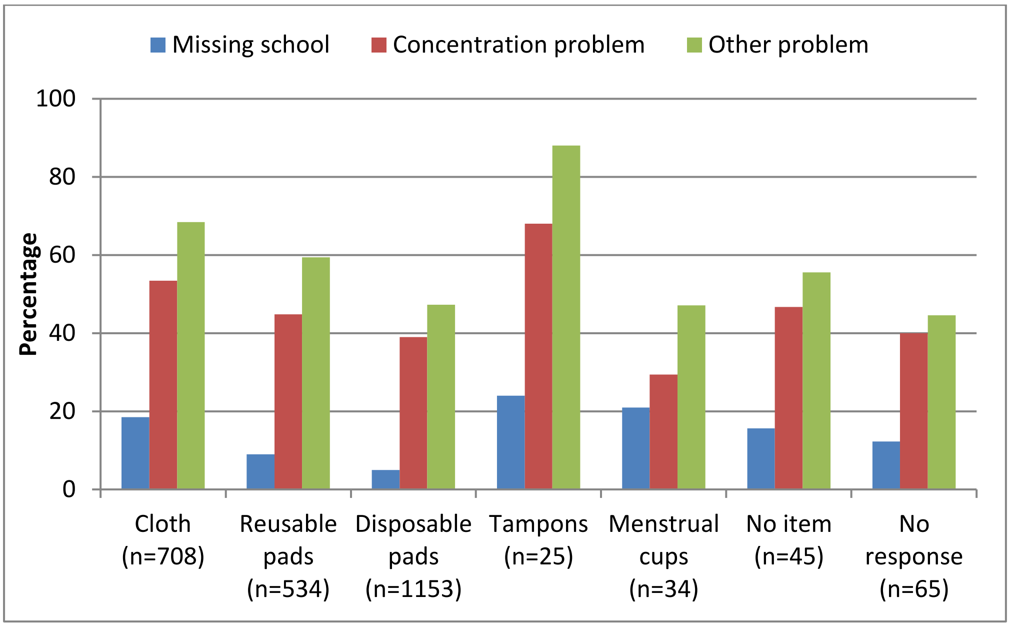

P-values for comparisons by MHM item for outcome "missing school during menstruation"

	**Cloth**	**Reusable pads**	**Disposable pads**	**Tampons**	**Cups**
**Cloth**	X				
**Reusable pads**	<0.001	X			
**Disposable pads**	<0.001	0.005	X		
**Tampons**	0.572	0.039	<0.001	X	
**Cups**	0.595	0.068	<0.001	0.755	X

P-values for comparisons by MHM item for outcome "concentration problems during menstruation"

	**Cloth**	**Reusable pads**	**Disposable pads**	**Tampons**	**Cups**
**Cloth**	X				
**Reusable pads**	0.007	X			
**Disposable pads**	<0.001	0.033	X		
**Tampons**	0.316	0.070	0.013	X	
**Cups**	0.024	0.195	0.268	0.012	X

P-values for comparisons by MHM item for outcome "other problems during menstruation"

	**Cloth**	**Reusable pads**	**Disposable pads**	**Tampons**	**Cups**
**Cloth**	X				
**Reusable pads**	0.001	X			
**Disposable pads**	0.001	<0.001	X		
**Tampons**	0.105	0.016	<0.001	X	
**Cups**	0.023	0.356	0.975	0.005	X

FIGURE S3: SPECIFICATION OF SCHOOL PROBLEMS RELATED TO MENSTRUATION BY TYPE OF MENSTRUAL ITEM USED, THREE STATES IN INDIA, 2015

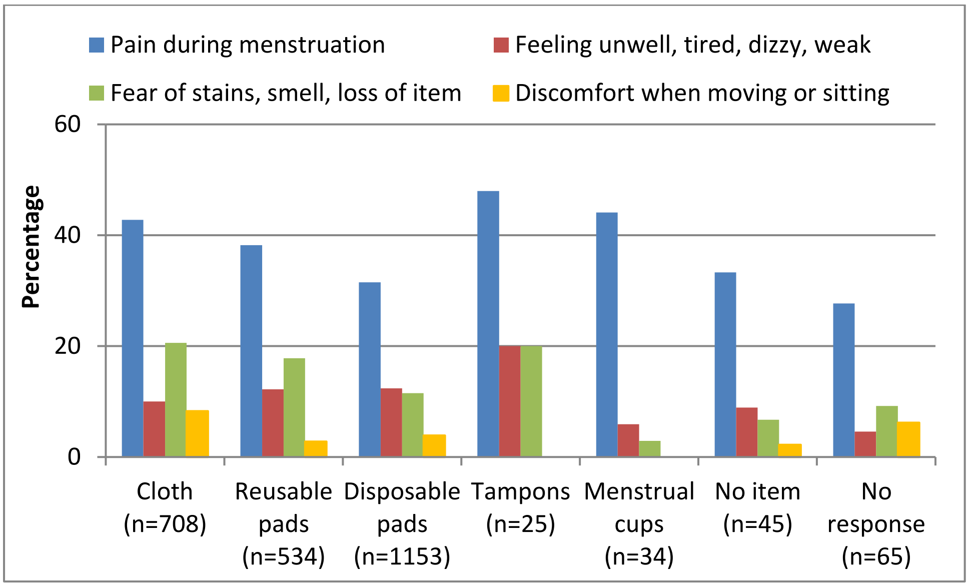

P-values for comparisons by MHM item for outcome “Pain during menstruation”

	Cloth	Reusable pads	Disposable pads	Tampons	Cups
**Cloth**	X				
**Reusable pads**	0.103	X			
**Disposable pads**	<0.001	0.007	X		
**Tampons**	0.605	0.325	0.079	X	
**Cups**	0.879	0.492	0.119	0.767	X

P-values for comparisons by MHM item for outcome “Feeling unwell, tired, dizzy, weak”

	Cloth	Reusable pads	Disposable pads	Tampons	Cups
**Cloth**	X				
**Reusable pads**	0.231	X			
**Disposable pads**	0.119	0.894	X		
**Tampons**	0.108	0.248	0.257	X	
**Cups**	0.428	0.270	0.253	0.098	X

P-values for comparisons by MHM item for outcome “Fear of stains, smell, and loss of item”

	Cloth	Reusable pads	Disposable pads	Tampons	Cups
**Cloth**	X				
**Reusable pads**	0.212	X			
**Disposable pads**	<0.001	<0.001	X		
**Tampons**	0.940	0.778	0.193	X	
**Cups**	0.012	0.025	0.119	0.032	X

P-values for comparisons by MHM item for outcome “Discomfort when moving or sitting”

	Cloth	Reusable pads	Disposable pads	Tampons	Cups
**Cloth**	X				
**Reusable pads**	<0.001	X			
**Disposable pads**	<0.001	0.259	X		
**Tampons**	0.132	0.396	0.314	X	
**Cups**	0.079	0.322	0.240	0% *vs.* 0%	X

FIGURE S4: PREFERRED MHM ITEM BY ITEM CURRENTLY USING, GOVERNMENT SCHOOLS IN THREE STATES IN INDIA, 2015

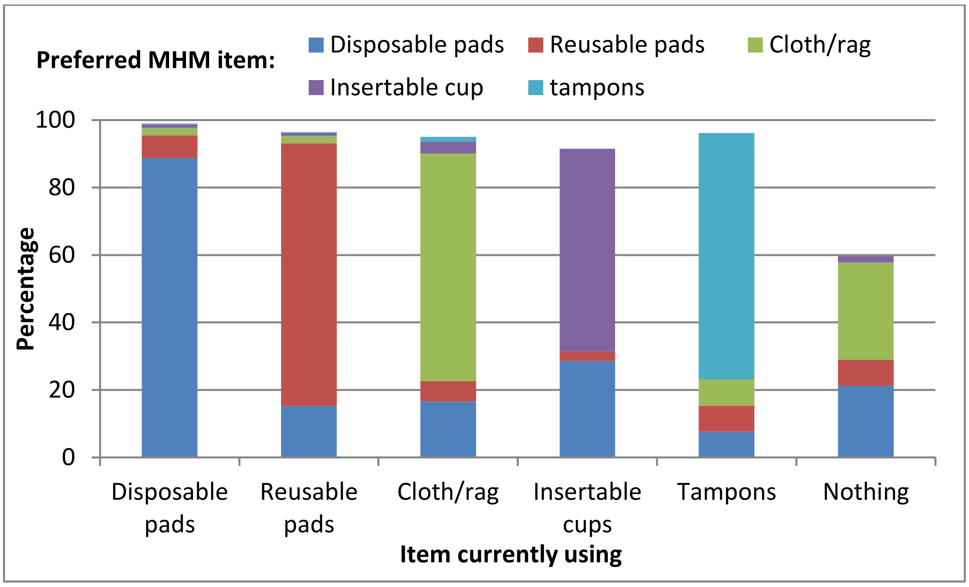

TABLE S1: SUMMARY TABLE OVERVIEWING POPULATION, ECONOMIC, EDUCATIONAL, HEALTH AND SANITATION INDICATORS IN STATES INVOLVED AND THE WHOLE OF INDIA

**2011 census***	**Chhattisgarh**	**Tamil Nadu**	**Maharashtra**	**All India**
Districts	27	32	36	[28 states]
Area	135,192 sq. km.	130,060 sq. km.	307,713 sq. km	
Population	25,545,198	72,147,030	112,374,333	1.21 billion
Pop density	189 per sq km	555 per sq km	365 per sq km	382 per sq km
Literacy	70% (F 60% M 80%)	80% (F 73% M 87%)	82% (F 70% M 88%)	74.0%
% rural	76.8%	51.6%	54.8%	
F:M sex ratio	991:1,000	996:1000	929:1000	940:1000
Human development index †	0.45	0.54	0.55	0.50
Mean years school[2]	3.39	4.79	5.12	4.10
Poverty ratio (2013)	39.9%	11.3%	17.4%	21.9%
Language	Chhattisgarhi (Hindi)	Tamil	Marathi	-
Scheduled tribes	34%	1.18%	8.9%	
Main tribes	*Gond,Halbi, Halba, Kamar/Bujia, Oraon.*	-	*Adivasis, Thakar, Warli, Konkana,Halba*	-
Scheduled castes	12%	21.5%	11.8%	-
Number with no bathrooms per 1000 households‡	Rural 849<br>Urban 349	Rural 577<br>Urban 155	Rural 542<br>Urban 179	Rural 623<br>Urban 167
Number with no latrines per 1000 households	Rural 767<br>Urban 249	Rural 664<br>Urban 122	Rural 540<br>Urban 69	Rural 594<br>Urban 88
Number with garbage disposals per 1000 households	Rural 639<br>Urban 551	Rural 363<br>Urban 834	Rural 549<br>Urban 908	Rural 320<br>Urban 758
P/UpP schools with drinking water§	98.5%	100%	99.4%	97.8%
P/UpP schools with girls toilet facility (all)	90.1%	97.4%	98.7%	91.3%
P/UpP schools with medical check ups	75.6%	95.6%	93.5%	73.0%
Girls dropout Class I-X**	51.4%	35.0%	38.8%	52.2%
Female teachers to 100 male (high/secondary)	65	130	58	66
Pupil to teacher ratio (high/ secondary)	43	35	32	32

Abbreviations: F: female, M: male, P/UpP: primary/upper primary (all government schools)
* http://www.census2011.co.in/census/state
† UNDP inequality adjusted human development index for India's states 2011
‡ NSS KI (69/1.2): Key indicators of Drinking Water, Sanitation, Hygiene and Housing Condition in India, GoI July-December 2012
§ District Information System for Education, http://www.dise.in/AR.htm Analytic Tables 2013-14
** http://mhrd.gov.in/statist?field_statistics_category_tid=33

TABLE S2: GIRLS' BEHAVIOUR WITH REGARDS TO CULTURAL TABOOS AND RESTRICTIONS DURING MENSTRUATION BY STATE AND SCHOOL TYPE, INDIA 2015

	Maharashtra		Chhattisgarh		Tamil Nadu		All 3 states
	**Regular school n (%)**	**Model school n (%)**	**Regular school N (%)**	**Model school n (%)**	**Regular school n (%)**	**Model school n (%)**	**Total**
	N=664	N=173	N=691	N=236	N=717	N=83	N=2564
Religious restrictions[1]							
No	33 (5.0)	54 (31.2)	62 (9.0)	12 (5.1)	40 (5.6)	20 (24.1)	221 (8.6)
Yes	627 (94.3)	110 (63.6)	591 (85.5)	209 (88.6)	665 (92.8)	63 (75.9)	2265 (88.3)
No response	4 (0.6)	9 (5.2)	38 (5.5)	15 (6.4)	12 (1.7)	0	78 (3.0)
Different sleeping arrangements[2*]							
Yes	81 (12.2)	20 (11.6)	127 (18.4)	24 (10.2)	269 (37.5)	15 (18.1)	536 (20.9)
No	568 (85.5)	148 (85.6)	516 (74.7)	199 (84.3)	436 (60.8)	68 (81.9)	1935 (75.5)
No response	15 (2.3)	5 (2.9)	48 (7.0)	13 (5.5)	12 (1.7)	0	93 (3.6)
Different behaviour in house[3*]							
Yes	73 (11.0)	32 (18.5)	84 (12.2)	16 (6.8)	179 (25.0)	15 (18.1)	399 (15.6)
No	580 (87.4)	132 (76.3)	572 (82.8)	209 (88.6)	523 (72.9)	68 (81.9)	2084 (81.3)
No response	11 (1.7)	9 (5.2)	35 (5.1)	11 (4.7)	15 (2.1)	0	81 (3.2)
Different behaviour outside house[4*]							
Yes	60 (9.0)	14 (8.1)	93 (13.5)	22 (9.3)	110 (15.3)	8 (9.6)	307 (12.0)
No	593 (89.3)	149 (86.1)	554 (80.2)	203 (86.0)	598 (83.4)	75 (90.4)	2172 (84.7)
No response	11 (1.7)	10 (5.8)	44 (6.4)	11 (4.7)	9 (1.3)	0	85 (3.3)
Eat/cook different[5*]							
Yes	25 (3.8)	20 (11.6)	40 (5.8)	10 (4.2)	72 (10.0)	19 (22.9)	186 (7.3)
No	630 (94.9)	147 (85.0)	624 (90.3)	214 (90.7)	638 (89.0)	62 (74.7)	2315 (90.3
No response	9 (1.4)	6 (3.5)	27 (3.9)	12 (5.1)	7 (1.0)	2 (2.4)	63 (2.5)
Can exercise[5]							
Yes	58 (8.7)	81 (46.8)	104 (15.1)	25 (10.6)	50 (7.0)	24 (28.9)	342 (13.3)
No	591 (89.0)	86 (49.7)	542 (78.4)	197 (83.5)	658 (91.8)	57 (68.7)	2131 (83.1)

No response	15 (2.3)	6 (3.5)	45 (6.5)	14 (5.9)	9 (1.3)	2 (2.4)	91 (3.6)

[1]P<0.05 comparing model schools vs. regular schools, and in Maharashtra and Tamil Nadu model vs. regular school

[2]P<0.05 comparing by state, model schools vs. regular schools, and in Chhattisgarh and Tamil Nadu model vs. regular school

[3]P<0.05 comparing by state, and in Maharashtra model vs. regular school

[4]P<0.05 comparing by state, model vs. regular school and in Maharashtra model vs. regular school

[5]P<0.05 comparing model schools vs. regular schools, by state, and in Maharashtra and Tamil Nadu model vs. regular school

*Different sleeping arrangements at home: *e.g.* girls reported to be banned to separate rooms or outside the house (veranda) during their menstruation.

Different behaviour in the house: *e.g.* less interactions with other household members, not getting close to or touch them, having to sit at a different place from them during the period, feeling irritated, being banned to outside the house, and being left alone.

Different behaviour outside the house: *e.g.* less outside playing and keeping distance from relatives, friends, and men.

Eating or cooking food differently: *e.g.* eating mainly sweet foods during the period or eating more green vegetables or not eating sweets during menstruation, not cooking or touching certain food, using and washing their own utensils, not preparing meals during period, or eating healthy food or foods with more iron content.

TABLE S3: MENSTRUAL HYGIENE ITEM USED AND PREFERENCE BY ADOLESCENT SCHOOL GIRLS IN THREE STATES IN INDIA, 2015

	**Maharashtra**				**Chhattisgarh**				**Tamil Nadu**				**All 3 states**	
	**Regular school**		**Model school**		**Regular school**		**Model school**		**Regular school**		**Model school**		**Total**	
	N=664		N=173		N=691		N=236		N=717		N=83		N=2564	
Items for MHM[1]	Using now n (%)	Likes to use (%)	Using now n (%)	Likes to use (%)	Using now n (%)	Likes to use (%)	Using now n (%)	Likes to use (%)	Using now n (%)	Likes to use (%)	Using now n (%)	Likes to use (%)	Using now n (%)	Likes to use (%)
Nothing	4 (0.6)	(0.8)	2 (1.2)	(1.2)	15 (2.2)	(2.6)	3 (1.3)	(0.4)	20 (2.8)	(2.9)	1 (1.2)	0	45 (1.8)	(1.8)
Cloth/rag	76 (11.5)	(9.0)	28 (16.2)	(8.7)	469 (67.9)	(50.5)	97 (41.1)	(32.2)	34 (4.7)	(5.0)	4 (4.8)	(6.0)	708 (27.6)	(21.1)
Reusable pads	251 (37.8)	(36.5)	56 (32.4)	(36.4)	34 (4.9)	(5.6)	24 (10.2)	(13.1)	152 (21.2)	(21.8)	17 (20.5)	(19.3)	534 (20.8)	(21.3)
Disposable pads	311 (46.8)	(49.9)	81 (46.8)	(46.2)	144 (20.8)	(31.8)	87 (36.9)	(44.5)	472 (65.8)	(64.0)	58 (70.0)	(71.1)	1153 (45.0)	(48.9)
Tampons	9 (1.4)	(1.2)	1 (0.6)	(1.2)	4 (0.6)	(1.3)	2 (0.9)	(1.3)	8 (1.1)	(1.3)	1 (1.2)	(2.4)	25 (1.0)	(1.3)
Menstrual cup	1 (0.2)	(0.6)	0	0	5 (0.7)	(3.3)	6 (2.5)	(5.1)	20 (2.8)	(3.2)	2 (2.4)	(1.2)	34 (1.3)	(2.5)
No response	12 (1.8)	(2.1)	5 (2.9)	(6.4)	20 (2.9)	(4.8)	17 (7.2)	(3.4)	11 (1.5)	(1.8)	0	0	65 (2.5)	(3.1)

[1]"What do you use now" and "What do you like to use": P<0.05 by state, and in Chhattisgarh model vs. regular school

TABLE S4: OTHER FACILITATORS BY SCHOOLS OF MENSTRUAL HYGIENE MANAGEMENT, GOVERNMENT SCHOOLS IN INDIA, 2015

	Maharashtra		Chhattisgarh		Tamil Nadu		All 3 states
	Regular school n (%)	Model school n (%)	Regular school N (%)	Model school n (%)	Regular school n (%)	Model school n (%)	Total
	N=664	N=173	N=691	N=236	N=717	N=83	N=2564
Toilets clean at last visit[1]							
Yes	417 (62.8)	143 (82.7)	265 (38.4)	72 (30.5)	536 (74.8)	83 (100.0)	1516 (59.1)
No	231 (34.8)	20 (11.6)	369 (53.4)	153 (64.8)	176 (24.6)	0	949 (37.0)
No response	16 (2.4)	10 (5.8)	57 (8.3)	11 (4.7)	5 (0.7)	0	99 (3.9)
Are there reasons girls to go outside of school for toilet needs rather than use school toilets?[2]							
Yes†	115 (17.3)	34 (19.7)	206 (29.8)	31 (13.1)	82 (11.4)	4 (4.8)	472 (18.4)
No	518 (78.0)	126 (72.8)	386 (55.9)	173 (73.3)	611 (85.2)	78 (94.0)	1892 (73.8)
Other	5 (0.8)	0	3 (0.4)	0 (0.0)	1 (0.1)	0	9 (0.4)
No response	26 (3.9)	13 (7.5)	96 (13.9)	32 (13.6)	23 (3.2)	1 (1.2)	191 (7.5)
Can you wash yourself in school when leaking?[3]							
Can always wash in school	230 (34.6)	131 (75.7)	317 (45.9)	114 (48.3)	443 (61.8)	72 (86.8)	1307 (51.0)
Can sometimes wash	47 (7.1)	12 (6.9)	113 (16.4)	50 (21.2)	209 (29.2)	11 (13.3)	442 (17.2)
Can never wash in school	375 (56.5)	18 (10.4)	219 (31.7)	60 (25.4)	48 (6.7)	0	720 (28.1)
No response	12 (1.8)	12 (6.9)	42 (6.1)	12 (5.1)	17 (2.4)	0	95 (3.7)
What happens if you can't wash?*[2]	N=422	N=30	N=332	N=110	N=257	N=11	N=1162
Stay dirty until time to go home	131 (31.0)	11 (36.7)	138 (41.6)	43 (39.1)	71 (27.6)	1 (9.1)	395 (34.0)
Go home, then clean and return	159 (37.7)	13 (43.3)	93 (28.0)	16 (14.6)	133 (51.8)	8 (72.7)	422 (36.3)
Go home and stay home	130 (30.8)	6 (20.0)	82 (24.7)	49 (44.6)	45 (17.5)	1 (9.1)	313 (26.9)
No response	2 (0.5)	0	19 (5.7)	2 (1.8)	8 (3.1)	1 (9.1)	32 (2.8)
	N=664	N=173	N=691	N=236	N=717	N=83	N=2564
Are there good wash facilities for cleaning a cloth in school?[4]							
Yes	108 (16.3)	82 (47.4)	129 (18.7)	67 (28.4)	231 (32.2)	50 (60.2)	667 (26.0)
No	509 (76.7)	74 (42.8)	361 (52.2)	113 (47.9)	372 (51.9)	22 (26.5)	1451 (56.6)
Don't know	28 (4.2)	5 (2.9)	152 (22.0)	44 (18.6)	101 (14.1)	10 (12.1)	340 (13.3)
No response	19 (2.9)	12 (6.9)	49 (7.1)	12 (5.1)	13 (1.8)	1 (1.2)	106 (4.1)
Is there a point person in the school for menstrual problems?[4]							
Yes	298 (44.9)	106 (61.3)	261 (37.8)	147 (62.3)	431 (60.1)	73 (88.0)	1316 (51.3)
No	309 (46.5)	41 (23.7)	303 (43.9)	59 (25.0)	271 (37.8)	8 (9.6)	991 (38.7)
Don't know	36 (5.4)	9 (5.2)	84 (12.2)	17 (7.2)	0	0	146 (5.7)

No response	21 (3.2)	17 (9.8)	43 (6.2)	13 (5.5)	15 (2.1)	2 (2.4)	111 (4.3)
	N=298	N=106	N=261	N=147	N=431	N=73	N=1316
Point person is female teacher[4]	273 (91.6)	86 (81.1)	173 (66.3)	116 (78.9)	272 (63.1)	69 (94.5)	989 (75.2)
	N=664	N=173	N=691	N=236	N=717	N=83	N=2564
Disposal of MHM items at school							
Burn pit[1]	43 (6.5)	56 (32.4)	197 (28.5)	44 (18.6)	163 (22.7)	11 (13.3)	514 (20.1)
Buckets/dustbin[5]	28 (4.2)	4 (2.3)	106 (15.3)	26 (11.0)	232 (32.4)	3 (3.6)	399 (15.6)
Take back home[3]	272 (41.0)	19 (11.0)	129 (18.7)	50 (21.2)	61 (8.5)	0	531 (20.7)
Rubbish pit[1]	187 (28.2)	26 (15.0)	83 (12.0)	53 (22.5)	86 (12.0)	0	435 (17.0)
Down toilet/latrine[6]	16 (2.4)	1 (0.6)	29 (4.2)	8 (3.4)	141 (19.7)	12 (14.5)	207 (8.1)
School incinerator[4]	44 (6.6)	47 (27.2)	7 (1.0)	20 (8.5)	16 (2.2)	53 (63.9)	187 (7.3)
Other	23 (3.5)	0	2 (0.3)	0	2 (0.3)	2 (2.4)	29 (1.1)
No response	51 (7.7)	20 (11.6)	138 (20.0)	35 (14.8)	16 (2.2)	2 (2.4)	262 (10.2
Good disposal facilities in school[4]							
Yes	80 (12.1)	60 (34.7)	90 (13.0)	64 (27.1)	321 (44.8)	79 (95.2)	694 (27.1)
No	528 (79.5)	92 (53.2)	346 (50.1)	90 (38.1)	309 (43.1)	0	1365 (53.2)
Don’t know	29 (4.4)	6 (3.5)	175 (25.3)	69 (29.2)	71 (9.9)	1 (1.2)	351 (13.7)
No response	27 (4.1)	15 (8.7)	80 (11.6)	13 (5.5)	16 (2.2)	3 (3.6)	154 (6.0)
Provides pain relief when needed[4]							
Yes	54 (8.1)	73 (42.2)	67 (9.7)	61 (25.9)	222 (31.0)	56 (67.5)	533 (20.8)
No	598 (90.1)	90 (52.0)	591 (85.5)	166 (70.3)	481 (67.1)	25 (30.1)	1951 (76.1)
No response	12 (1.8)	10 (5.8)	33 (4.8)	9 (3.8)	14 (2.0)	2 (2.4)	80 (3.1)
Regularly giving pads[7]							
Yes	62 (9.3)	82 (47.4)	89 (12.9)	71 (30.1)	576 (80.3)	75 (90.4)	955 (37.3)
No	565 (85.1)	75 (43.4)	483 (69.9)	136 (57.6)	81 (11.3)	7 (8.4)	1347 (52.5)
Don’t know	18 (2.7)	3 (1.7)	72 (10.4)	20 (8.5)	44 (6.1)	0	157 (6.1)
No response	19 (2.9)	13 (7.5)	47 (6.8)	9 (3.8)	16 (2.2)	1 (1.2)	105 (4.1)

* Reasons mentioned: Bathroom not clear or no water

† Only among girls who said they can sometimes or never wash themselves in school

[1]P<0.05 for comparison by state, and within all states model vs. regular school

[2]P<0.05 for comparison by state, model vs. regular schools, and in Chhattisgarh model vs. regular school

[3]P<0.05 for comparison by state, model vs. regular school, and Maharashtra and Tamil Nadu model vs. regular school

[4]P<0.05 for comparison by state, model vs. regular school, and within all states model vs. regular school

[5]P<0.05 for comparison by state, model vs. regular school, and in Tamil Nadu model vs. regular school

[6]P<0.05 for comparison by state and model vs. regular school

[7]P<0.05 for comparison by state, model vs. regular school, and Maharashtra and Chhattisgarh vs. regular school

TABLE S5. EDUCATION ON MHM IN SCHOOLS, SURVEYS IN GOVERNMENT SCHOOLS IN THREE STATES IN INDIA, 2015

	**Maharashtra**		**Chhattisgarh**		**Tamil Nadu**		**All 3 states**
	Regular school n (%)	Model school n (%)	Regular school n (%)	Model school n (%)	Regular school n (%)	Model school n (%)	Total
	N=664	N=173	N=691	N=236	N=717	N=83	N=2564
MHM taught in school[1]							
Yes	143 (21.5)	122 (70.5)	57 (8.3)	53 (22.5)	377 (52.6)	80 (96.4)	832 (32.5)
No	488 (73.5)	35 (20.2)	549 (79.5)	157 (66.5)	221 (30.8)	1 (1.2)	1451 (56.6)
Don’t know	17 (2.6)	3 (1.7)	46 (6.7)	15 (6.4)	107 (14.9)	1 (1.2)	189 (7.4)
No response	16 (2.4)	13 (7.5)	39 (5.6)	11 (4.7)	12 (1.7)	1 (1.2)	92 (3.6)
If yes, how is it taught?[2]	N=143	N=122	N=57	N=53	N=377	N=80	N=832
Hygiene lesson	36 (25.2)	71 (58.2)	27 (47.4)	30 (56.6)	249 (66.1)	65 (81.3)	478 (57.5)
Biology lesson	74 (51.8)	26 (21.3)	13 (22.8)	9 (17.0)	30 (8.0)	3 (3.8)	155 (18.6)
Outside person	25 (17.5)	22 (18.0)	7 (12.3)	7 (13.2)	84 (22.3)	10 (12.5)	155 (18.6)
Other	0	0	1 (1.8)	1 (1.9)	8 (2.1)	2 (2.5)	12 (1.4)
No response	8 (5.6)	3 (2.5)	9 (15.8)	6 (11.3)	6 (1.6)	0	32 (3.9)
Girls only lesson?[3]	N=143	N=122	N=57	N=53	N=377	N=80	N=832
Girls only	126 (88.1)	107 (87.7)	37 (64.9)	42 (79.3)	294 (78.0)	75 (93.8)	681 (81.9)
Boys too	13 (9.1)	8 (6.6)	9 (15.8)	2 (3.8)	77 (20.4)	5 (6.3)	114 (13.7)
No response	4 (2.8)	7 (5.7)	11 (19.3)	9 (17.0)	6 (1.6)	0	37 (4.6)
Written materials on MHM available?[4]							
Yes	54 (8.1)	117 (67.6)	39 (5.6)	32 (13.6)	163 (22.7)	72 (86.8)	477 (18.6)
No	508 (76.5)	37 (21.4)	439 (63.5)	131 (55.5)	371 (51.7)	7 (8.4)	1493 (58.2)
Don’t know	57 (8.6)	5 (2.9)	141 (20.4)	56 (23.7)	172 (24.0)	4 (4.8)	435 (17.0)
No response	45 (6.8)	14 (8.1)	72 (10.4)	17 (7.2)	11 (1.5)	0	159 (6.2)
Program in school about MHM[4]							
Yes	134 (20.2)	135 (78.0)	18 (2.6)	33 (14.0)	137 (19.1)	65 (78.3)	522 (20.4)
No	477 (71.8)	25 (14.5)	540 (78.2)	147 (62.3)	371 (51.7)	5 (6.0)	1565 (61.0)
Don’t know	30 (4.5)	7 (4.1)	83 (12.0)	33 (14.0)	197 (27.5)	13 (15.7)	363 (14.2)
No response	23 (3.5)	6 (3.5)	50 (7.2)	23 (9.8)	12 (1.7)	0 (0.0)	114 (4.5)

[1]P<0.05 for comparison by state, model vs. regular school overall and within states

[2]P<0.05 for comparison by state, and in Maharashtra model vs. regular school

[3]P<0.05 for comparison by state, model vs. regular school and in Tamil Nadu model vs. regular school

[4]P<0.05 for comparison by state, model vs. regular school, and within all states model vs. regular school

TABLE S6. THE ASSOCIATION BETWEEN SANITATION RELATED FACTORS AND MISSING SCHOOL DURING MENSTRUATION BY ADOLESCENT GIRLS, 3 STATES IN INDIA, 2015

Factor	Missing school: n/N (%)	Univariate analysis Prevalence ratio, 95% CI*	p	Multivariate analysis Prevalence ratio, 95% CI*	p
Enough toilets in school					
Yes	93/1240 (7.5)	0.55, 0.39-0.78	0.001		
No	156/1153 (13.5)	Reference			
Not reported	16/113 (14.2)	1.05, 0.52-2.09	0.898		
When can you use the toilet?					
Any time	100/887 (11.3)	Reference		Reference	
Only during breaks	106/1365 (7.8)	0.69, 0.49-0.96	0.029	0.72, 0.53-0.99	0.041
Other responses§	21/85 (24.7)	3.18, 1.70-5.95	<0.001	1.76, 1.05-2.96	0.033
No response	38/169 (22.5)	2.90, 1.91-4.38	<0.001	1.74, 1.23-2.47	0.002
Toilets clean					
Always clean	73 (1128 (6.5)	Reference		Reference	
Sometimes clean	122/978 (12.5)	1.93, 1.32-2.80	0.001	1.64, 1.16-2.33	0.006
Never clean or NR	70/396 (17.7)	2.73, 1.74-4.29	<0.001	1.93, 1.27-2.94	0.002
Toilets for girls					
For female staff & girls	60/582 (10.3)	1.40, 0.94-2.07	0.096	NS	
For girls only	68/921 (7.4)	Reference			
For boys and girls	79/549 (14.4)	1.95, 1.07-3.55	0.029		
For all staff & students	38/320 (11.9)	1.61, 0.96-2.71	0.073		
No response	20/134 (14.9)	2.02, 1.10-3.72	0.024		
Can wash in school					
Can always wash	95/1287 (7.4)	Reference		Reference	
Can sometimes wash	47/437 (10.8)	1.46, 1.07-1.98	0.017	1.34, 1.05-1.73	0.020
Can never wash or NR	123/778 (15.8)	2.14, 1.56-2.94	<0.001	1.71, 1.28-2.29	<0.001

*All analyses adjusted for school as cluster

§ Other included responses such as queuing before toilet, toilet unusable, no toilet present, go home for change
